# Comment on “Unveiling the Atmospheric Oxidation of Hexafluoroisobutylene, (CF_3_)_2_C=CH_2_, with Cl Atom, NO_3_ Radical, and O_3_ Molecule”

**DOI:** 10.1021/acs.jpca.5c02973

**Published:** 2025-06-25

**Authors:** Claus Jørgen Nielsen

**Affiliations:** Section for Environmental Sciences, Department of Chemistry, University of Oslo, P.O.Box. 1033, Blindern NO-0315, Oslo, Norway

## Introduction

In their paper titled “Unveiling the Atmospheric Oxidation of Hexafluoroisobutylene, (CF_3_)_2_C=CH_2_, with Cl Atom, NO_3_ Radical, and O_3_ Molecule”, Changmai et al.^1^ conducted investigations into the title reactions using Kohn–Sham density functional theory calculations.^2^ They employed the M06-2X functional^3^ and the 6-311++G­(d,p) basis set (hereafter abbreviated as BS). Additionally, they reported results from MP2/BS calculations, single point energies derived from CCSD­(T)/BS//M06-2X/BS calculations and reaction rate coefficients obtained through variational transition state theory^4^ in the high-pressure limit. This commentary addresses a systematic error in Changmai et al.’s studies of the NO_3_ radical reaction with (CF_3_)_2_C=CH_2_. The error does not change the overall conclusion of the study: the atmospheric chemical lifetime of (CF_3_)_2_C=CH_2_ is not affected by nitrate radical reactions.

The NO_3_ radical presents a computational challenge. Its electronic ground state has *D*_3*h*_ symmetry (X̃^2^A_2_^′^).^5,6^ The experimental NO distance is 1.238 Å (*r*_0_ structure),^7^ and the fundamental vibrational modes (in cm^–1^) are 1050 A_1_′, 762.3 A_2_″, 1492.4 E′ and 360 E′.^8^

It is not possible to calculate the electronic structure of the NO_3_ radical correctly using any standard size extensive UHF wave function based method that is also applicable to larger systems.^9^ HF calculations locate 3 distinct minimum energy structures: one of *D*_3*h*_ symmetry and two of *C*_2*v*_ symmetry having lower energies and respectively 2 short and 1 long NO distance (2s,l), and 1 short and 2 long NO distances (2l,s).^9^ In contrast, MP2 calculations place the *D*_3*h*_ structure lower in energy than the two *C*_3*v*_ structures; even CCSD­(T) cannot completely overcome the symmetry breaking of the reference function, and still three energy minima are located.^9^

There are many functionals developed for use in Kohn–Sham density functional theory calculations. Most of the commonly used “pure” functionals locate a single minimum energy NO_3_ structure of *D*_3*h*_ symmetry. Many hybrid functionals also predict the *D*_3*h*_ symmetry structure as the global energy minimum, but there are also many that show symmetry breaking. An early study found that the exchange functional is more important than the correlation functional in resisting symmetry breaking and that mixing large fractions of Hartree–Fock exchange with other functionals can cause symmetry breaking.^10^ The M06-2X global hybrid functional, having 54% HF exchange,^3^ is among those locating the NO_3_ *D*_3*h*_-structure as a saddle point.

## (CF_3_)_2_C=CH_2_ + NO_3_ Radical Reaction

There are three routes in the (CF_3_)_2_C=CH_2_ + NO_3_ reaction. Following Changmai et al.,^1^ the routes are labeled [Disp-formula eqR4] (C^2^-addition), [Disp-formula eqR5] (C^1^-addition) and [Disp-formula eqR6] (H-abstraction).

R4 $${\left({\mathrm{CF}}_{3}\right)}_{2}C={\mathrm{CH}}_{2}+{\mathrm{NO}}_{3}\to {\left({\mathrm{CF}}_{3}\right)}_{2}C\left({\mathrm{ONO}}_{2}\right)\text{−}ĊH_{2}$$

R5 $${\left({\mathrm{CF}}_{3}\right)}_{2}C={\mathrm{CH}}_{2}+{\mathrm{NO}}_{3}\to {\left({\mathrm{CF}}_{3}\right)}_{2}Ċ\text{−}{\mathrm{CH}}_{2}\left({\mathrm{ONO}}_{2}\right)$$

R6 $${\left({\mathrm{CF}}_{3}\right)}_{2}C={\mathrm{CH}}_{2}+{\mathrm{NO}}_{3}\to {\left({\mathrm{CF}}_{3}\right)}_{2}C=ĊH+{\mathrm{HNO}}_{3}$$

Changmai et al. located the barrier to [Disp-formula eqR6] around 14 kcal mol^–1^ above those of [Disp-formula eqR4] and [Disp-formula eqR5], and this route will not be addressed in the following.

The NO_3_ radical structures and vibrational frequencies obtained from M06-2X/BS calculations exhibit significant deviations from the experimental data. Although the Changmai et al. study does not explicitly report the NO_3_ radical structure, the vibrational frequencies listed in their Supporting Information (Table S1: 1673, 1381, 835, 809, 687, and 339 cm^–1^) indicate that a C_2*v*_^2s,l^ structured NO_3_ radical was selected as the reactant.

Assuming that the quantum chemistry method employed is consistent with experimental thermochemistry data, one can estimate the error in the calculated ground state NO_3_ radical electronic energy by integrating the theoretical method results for the NO_3_ radical formation reaction, N_2_ + 3O_2_(^3^Σ_g_) → 2NO_3_, the experimental standard enthalpy of formation for NO_3_ at 0 K (Δ_f_ *H* ^o^ = 18.97 ± 0.05 kcal mol^–1^),^11^ and the experimental fundamental modes of vibration for NO_3_.^8^

1 $$E_{\mathrm{elec}}\left(NO_{3}\right)=\Delta_{f}H^{{}^{\circ}}\left({\mathrm{NO}}_{3}\right)+\frac{1}{2}\left\{3E_{v=0}\left(O_{2}\right)+E_{v=0}\left(N_{2}\right)\right\}-E_{\mathrm{ZPE}}^{\exp}\left({\mathrm{NO}}_{3}\right)$$

The error introduced by disregarding vibrational anharmonicity in this approach is of the same order of magnitude as the experimental uncertainty in Δ_f_ *H* ^o^. The error introduced by assuming the quantum chemistry method being consistent with experimental thermochemistry data varies across different methods.^3^

Based on the results obtained in M06-2X/BS calculations for N_2_ and O_2_, the ground state electronic energy of the NO_3_ radical is estimated to be −280.197356 hartree; see Table 1S in the Supporting Information. This value is 10.62 kcal mol^–1^ lower than the *E*_elec_(NO_3_) utilized in the title study.

At the CCSD­(T)/BS//M06-2X/BS level of theory, the electronic energy of the *C*_2*v*_^2s,l^ structured NO_3_ radical is reported by Changmai et al. to be −279.679637 hartree. At this level of theory, the ground state electronic energy of the NO_3_ radical is estimated to be −279.696684 hartree (Table S1), which is 10.31 kcal mol^–1^ lower than the electronic energy used in the title study.

The energy profile presented for the (CF_3_)_2_C=CH_2_ + NO_3_ reaction in the title study is obviously not correct. The NO_3_ radical reaction occurs on a path that commences with a ground state NO_3_ radical having *D*_3*h*_-symmetry. The radical subsequently traverses saddle points in which it distorts toward (2s,l)-like structures, terminating in either HNO_3_ or in O_2_NO-Ṙ radicals. The path connecting the electronic ground state of the free NO_3_ radical and the (2s,l) distorted NO_3_ radical near saddle points to the reaction cannot be accurately described in M06-2X calculations. Furthermore, the postulated prereaction complexes (CR4 and CR6) are artifacts of the quantum chemistry methodology. Lastly, assuming that the saddle points to the reaction (TS4 and TS5) are reasonably accurately described in M06-2X calculations, these barriers are, in reality, 10–11 kcal mol^–1^ higher than reported.

Figure 1 illustrates how the alignment of the CCSD­(T)/BS//M06-2X/BS results to experimental thermochemistry data alters the potential energy landscape. The underlying quantum chemistry data (energies, corrected energies, Cartesian coordinates, vibrational frequencies, and rotational constants) are collected in Table S2. The vibrational frequencies compare well with those reported in the title paper. However, the present CCSD­(T)/BS//M06-2X/BS energies differ slightly due to implementation of stringent convergence criteria in the present geometry optimization.

**Figure 1:**
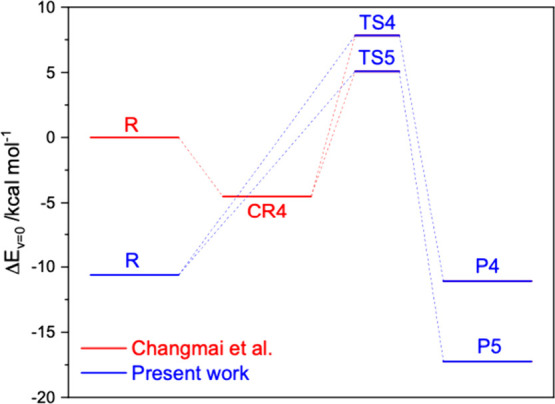
Relative energies of stationary points on the potential energy surface of the (CF_3_)_2_C=CH_2_ + NO_3_ reaction. Comparison of present results to those reported by Changmai et al.^1^

B3LYP,^12,13^ widely recognized as one of the most prominent functionals developed, has been extensively utilized in nearly all chemical domains. While its general performance in thermochemistry and kinetics is inferior to that of M06-2X,^3^ it stands out in identifying a single, *D*_3*h*_-structured NO_3_ radical as the global energy minimum. Furthermore, the B3LYP/BS calculations on O_2_, N_2_ and NO_3_ align with the experimental Δ_f_ *H* ^o^ at 0 K within 1.1 kcal mol^–1^. However, the vibrational frequencies obtained in B3LYP/BS calculations (1131 A1′, 801 A2″, 1108 and 284 E′) exhibit significant discrepancies with the experimental data. Notably, the E′-modes are calculated at lower wavenumbers compared to the experimental observations, and replacing the calculated *E*_ZPE_ (0.010743 hartree) by the experimental value (0.012569 hartree) brings B3LYP/BS calculations on O_2_, N_2_ and NO_3_ in perfect alignment with the thermochemistry data.

Given the suboptimal performance of B3LYP in thermochemical kinetics, B3LYP calculations are frequently “improved” in CCSD­(T) single-point calculations. This introduces an electronic energy of the NO_3_ radical that is discrepant with thermochemistry data, necessitating the application of eq 1.

Results from B3LYP/BS and CCSD­(T)/BS//B3LYP/BS calculations on the (CF_3_)_2_C=CH_2_ + NO_3_ reaction are included in Table S2. The calculated electronic energy for NO_3_, −279.683578 hartree, obtained from CCSD­(T)/BS//B3LYP/BS, is 7.37 kcal mol^–1^ higher than the estimated value from the radical formation reaction (−279.698518 hartree, Table S1). By substitution of both *E*_ZPE_(B3LYP/BS) and *E*_elec_(CCSD­(T)/BS//B3LYP/BS) by the experimental *E*_ZPE_ and the estimated *E*_elec_, the initially mismatched CCSD­(T)/BS//B3LYP/BS and CCSD­(T)/BS//M06-2X/BS results are reconciled, as demonstrated in Figure 2.

**Figure 2:**
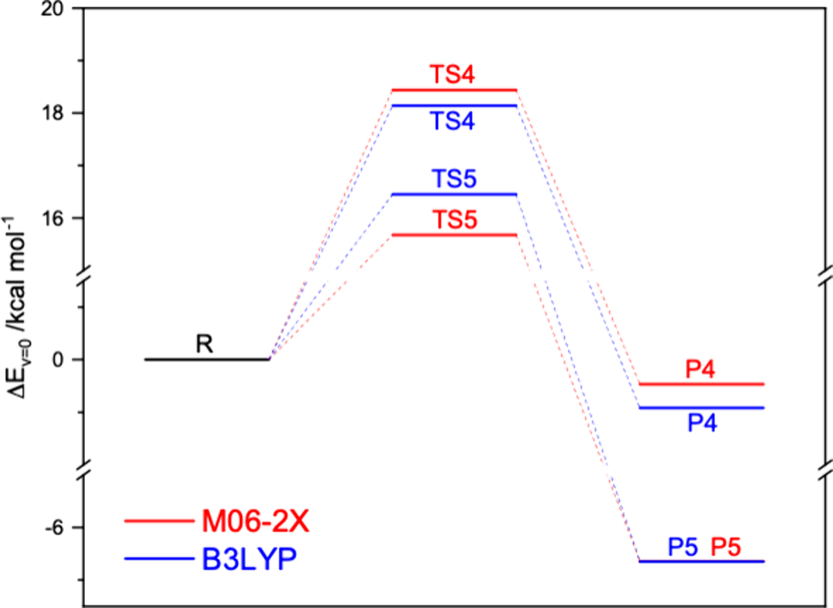
Comparison of stationary points on the potential energy surface of the (CF_3_)_2_C=CH_2_ + NO_3_ reaction obtained from CCSD­(T)/6-311++G­(d,p)//M06-2X/6-311++G­(d,p) and CCSD­(T)/6-311++G­(d,p)//B3LYP/6-311++G­(d,p) calculations. The underlying quantum chemistry data are listed in Tables S1 and S2.

Figure 2 illustrates the appropriateness of the present approach for correcting the electronic energies obtained in calculations on NO_3_ radical reactions by employing symmetry breaking methodologies. It is evident that the activation energies for the (CF_3_)_2_C=CH_2_ + NO_3_ addition reaction are significantly higher than those reported by Changmai et al., resulting in correspondingly lower rate coefficients. A simple extrapolation, based on the barriers indicated in Figure 2, suggests that the rate coefficient for the reaction is at least 5 orders of magnitude smaller than that presented by Changmai et al. Nevertheless, this does not alter the conclusion of the title study: the atmospheric chemical lifetime of (CF_3_)_2_C=CH_2_ is not affected by nitrate radical reactions.

## Conclusions

The NO_3_ radical cannot be accurately described in the M06-2X and CCSD­(T) calculations. Neither the electronic energies nor the zero-point energies align with experimental data. However, the error in the electronic energies can be estimated by integrating the theoretical method results for the NO_3_ radical formation reaction, N_2_ + 3O_2_(^3^Σ_g_) → 2NO_3_, the experimental standard enthalpy of formation for NO_3_ at 0 K, and the experimental fundamental modes of vibration for the NO_3_ radical.

## Supplementary Material



## Notes

Results from M06-2X, B3LYP, CCSD­(T)//M06-2X and CCSD­(T)//B3LYP calculations employing the 6-311++G­(d,p) basis set. Table S1: Electronic and zero-point energies from calculations on N_2_ and O_2_(^3^Σ_g_). Table S2: Results from calculations on the (CF_3_)_2_C=CH_2_ + NO_3_ reaction including Cartesian coordinates, vibrational frequencies and rotational constants (PDF)

## References

[1] Changmai R. R., Daimari S. R., Sarma M. (2025). Unveiling the Atmospheric Oxidation
of Hexafluoroisobutylene, (CF_3_)_2_C=CH_2_, with Cl Atom, NO_3_ Radical, and O_3_ Molecule. J. Phys. Chem. A.

[2] Kohn W., Sham L. J. (1965). Self-Consistent
Equations Including Exchange and Correlation
Effects. Phys. Rev..

[3] Zhao Y., Truhlar D. G. (2008). The M06 suite of
density functionals for main group
thermochemistry, thermochemical kinetics, noncovalent interactions,
excited states, and transition elements: two new functionals and systematic
testing of four M06-class functionals and 12 other functionals. Theor. Chem. Acc..

[4] Truhlar D. G., Garrett B. C. (1980). Variational transition-state theory. Acc. Chem. Res..

[5] Ishiwata T., Tanaka I., Kawaguchi K., Hirota E. (1985). Infrared diode laser
spectroscopy of the NO_3_ ν_3_ band. J. Chem. Phys..

[6] Kawaguchi K., Hirota E., Ishiwata T., Tanaka I. (1990). A reinvestigation of
the NO_3_ 1492 cm^-1^ band. J. Chem. Phys..

[7] Kawaguchi K., Ishiwata T., Hirota E., Tanaka I. (1998). Infrared spectroscopy
of the NO_3_ radical. Chem. Phys..

[8] Jacox M. E. (2003). Vibrational
and electronic energy levels of polyatomic transient molecules. Supplement
B. J. Phys. Chem. Ref. Data.

[9] Eisfeld W., Morokuma K. (2000). A detailed study on
the symmetry breaking and its effect
on the potential surface of NO_3_. J. Chem. Phys..

[10] Sherrill C. D., Lee M. S., Head-Gordon M. (1999). On the performance
of density functional
theory for symmetry-breaking problems. Chem.
Phys. Lett..

[11] Ruscic B., Pinzon R. E, Laszewski G. v., Kodeboyina D., Burcat A., Leahy D., Montoy D., Wagner A. F (2005). Active Thermochemical
Tables: thermochemistry for the 21st century. Journal of Physics: Conference Series.

[12] Lee C., Yang W., Parr R. G. (1988). Development of the Colle-Salvetti
correlation-energy formula into a functional of the electron density. Phys. Rev. B.

[13] Becke A. D. (1993). Density-functional
thermochemistry. III. The role of exact exchange. J. Chem. Phys..

