# Supplementary material for: Comment on “Unveiling the Atmospheric Oxidation of Hexafluoroisobutylene, (CF3)2C=CH2, with Cl Atom, NO3 Radical, and O3 Molecule”
Source: J Phys Chem A. 2025 Jun 25;129(26):5920–2. doi: 10.1021/acs.jpca.5c02973 (PMC12235629; doi:10.1021/acs.jpca.5c02973)
Supplement: Supplementary file 1 [file jp5c02973_si_001.pdf]

Comment on ´´Unveiling the Atmospheric Oxidation of Hexafluoroisobutylene, $(CF_3)_2C{=}CH_2$, with Cl Atom, NO_3 Radical, and O_3 Molecule´´ Changmai et al., J. Phys. Chem. A, 2025, 129, 3906-3920.

Claus Jørgen Nielsen

Section for Environmental Sciences, Department of Chemistry, University of Oslo, P.O.Box. 1033 – Blindern, NO-0315 Oslo, Norway.

Supplementary Information:

Table S1. QCC results for O_2 and N_2, and estimated energies of the NO_3 radical.
Electronic and zero-point energies (/Hartree) from calculations employing the 6-311++G(d,p) basis set.

	$O_2(^3\Sigma_g)$		N_2		NO_3	
QC Method	E_{elec}	E_{ZPE}	E_{elec}	E_{ZPE}	$E_{v=0}$[a]	E_{elec}[b]
M06-2X	-150.308589	0.004026	-109.522107	0.005756	-280.184786	-280.197356
CCSD(T)//M06-2X	-150.044124	0.004026	-109.314159	0.005756	-279.684115	-279.696684
B3LYP	-150.370417	0.003721	-109.559694	0.005570	-280.296872	-280.309442
CCSD(T)//B3LYP	-150.044814	0.003721	-109.314658	0.005570	-279.685949	-279.698518

[a] Estimated from equation (1) in the main text. [b] Calculated assuming $E_{ZPE}(NO_3)$ = 0.012569 Hartree.

Table S2. QCC results for the $(CF_3)_2C{=}CH_2$ + NO_3 reaction.
Electronic and zero-point energies (/Hartree), and relative energies including Zero Point Energies, $\Delta E_{v=0}$ (/kcal mol^{-1}).

Species	M06-2X/6-311++G(d,p)			CCSD(T)/6-311++G(d,p)	
	E_{Elec}	E_{ZPE}	$\Delta E_{v=0}$	E_{Elec}	$\Delta E_{v=0}$
$(CF_3)_2C{=}CH_2$	-752.662283	0.063570		-751.331541	
NO_3 (*2s,l*)	-280.180426	0.013043		-279.680251	
Sum reactants	-1032.842709	0.076612	0.00	-1031.011791	0.00
TS4	-1032.838205	0.078117	3.77	-1031.000815	7.83
P4	-1032.872112	0.078932	-17.00	-1031.031761	-11.08
TS5	-1032.842490	0.078668	1.43	-1031.005774	5.07
P5	-1032.886854	0.081959	-24.35	-1031.044628	-17.25
---*corrected*---*corrected*---*corrected*---*corrected*---*corrected*---*corrected*---					
$(CF_3)_2C{=}CH_2$	-752.662283	0.063570		-751.331541	
NO_3*	-280.197356	0.012569		-279.696684	
Sum reactants	-1032.859638	0.076139	0.00	-1031.028225	0.00
TS4	-1032.838205	0.078117	14.69	-1031.000815	18.44
P4	-1032.872112	0.078932	-6.07	-1031.031761	-0.47
TS5	-1032.842490	0.078668	12.35	-1031.005774	15.68
P5	-1032.886854	0.081959	-13.43	-1031.044628	-6.64

	B3LYP/6-311++G(d,p)			CCSD(T)/6-311++G(d,p)	
	E_{Elec}	E_{ZPE}	$\Delta E_{v=0}$	E_{Elec}	$\Delta E_{v=0}$
$(CF_3)_2C{=}CH_2$	-752.907119	0.061726		-751.331752	
NO_3 (D_{3h})	-280.309326	0.010537		-279.683578	
Sum reactants	-1033.216444	0.072263	0.00	-1031.015330	0.00
TS4	-1033.197362	0.075119	13.77	-1031.002191	10.04
P4	-1033.215281	0.075656	2.86	-1031.033101	-9.02
TS5	-1033.207463	0.075739	7.82	-1031.005508	8.35
P5	-1033.234758	0.078547	-7.55	-1031.045124	-14.75
---corrected---corrected---corrected---corrected---corrected---corrected---					
$(CF_3)_2C{=}CH_2$	-752.907119	0.061726		-751.331752	
NO_3 (D_{3h})*	-280.309326	0.012569		-279.698518	
Sum reactants	-1033.216444	0.074295	0.00	-1031.030270	0.00
TS4	-1033.197362	0.075119	12.49	-1031.002191	18.14
P4	-1033.215281	0.075656	1.58	-1031.033101	-0.92
TS5	-1033.207463	0.075739	6.54	-1031.005508	16.45
P5	-1033.234758	0.078547	-8.82	-1031.045124	-6.65

*Energies in red font have been corrected, see main text for details.

Table S2 continued. Vibrational frequencies (cm^{-1}), Rotational constants (GHz) and Cartesian coordinates (Å) of the species listed above.

Results from M06-2X/6-311++G(d,p) calculations	
$(CF_3)_2C{=}CH_2$<br><br>ν: 37.5, 62.8, 160.9, 192.5, 294.5, 316.8, 349.8, 358.6, 498.5, 500.7, 548.8, 548.9, 649.5, 686.9, 736.0, 769.3, 796.9, 893.9, 1035.0, 1116.1, 1203.6, 1207.3, 1244.4, 1276.1, 1285.1, 1419.6, 1457.5, 1772.2, 3193.5, 3290.7<br><br>B: 2.1525394, 1.0528961, 0.9382883	C 0.000000 0.686921 0.000000<br>C 0.000000 2.009565 0.000000<br>H 0.930185 2.562257 0.027010<br>H -0.930185 2.562257 -0.027010<br>C -1.278703 -0.111810 -0.002315<br>C 1.278703 -0.111810 0.002315<br>F -1.230867 -1.105584 -0.894330<br>F -2.330174 0.656580 -0.301545<br>F 1.513583 -0.659983 -1.194659<br>F 1.230868 -1.105582 0.894333<br>F 2.330174 0.656581 0.301542<br>F -1.513585 -0.659980 1.194660
NO_3 (*2s,l*)<br><br>ν: 339.5, 686.9, 809.0, 835.2, 1381.1, 1673.4<br>B: 14.2353015, 13.4155831, 6.9066460	N 0.000000 0.000000 -0.083788<br>O 0.000000 1.085170 -0.582690<br>O 0.000000 -1.085170 -0.582690<br>O 0.000000 0.000000 1.238695
TS4<br><br>ν: -296.6, 29.6, 45.7, 52.8, 66.8, 116.1, 139.7, 173.6, 208.0, 244.3, 295.2, 314.8, 342.5, 355.2, 463.1, 499.3, 545.1, 555.0, 594.7, 645.0, 650.4, 662.0, 693.2, 719.7, 797.1, 808.6, 896.8, 966.9, 1013.2, 1113.5, 1197.4, 1226.2, 1245.1, 1253.9, 1295.4, 1379.8, 1404.3, 1454.2, 1613.8, 1715.8, 3193.2, 3302.1,	C -0.469551 0.025462 0.531667<br>C -0.035396 -0.101176 1.812068<br>H 0.490952 0.710743 2.295107<br>H -0.157072 -1.035285 2.344347<br>C -1.410605 -1.015274 -0.055120<br>C -0.464460 1.401723 -0.111459<br>F -2.670705 -0.580650 0.079946<br>F -1.300826 -2.166751 0.609545

B: 0.9107228, 0.6044895, 0.4847672	F -1.474356 2.134331 0.373067<br>F -0.595445 1.349566 -1.428669<br>F 0.671209 2.039862 0.179457<br>F -1.202997 -1.256105 -1.339308<br>O 1.175305 -0.660029 -0.557418<br>N 2.426337 -0.472838 -0.086116<br>O 2.584214 -0.183663 1.069207<br>O 3.255470 -0.645343 -0.932531
P4<br><br>ν: 34.9, 43.0, 79.4, 101.6, 160.1, 176.6, 227.9, 240.9, 262.4, 286.0, 314.5, 328.6, 349.0, 359.6, 389.8, 503.7, 539.1, 546.8, 562.0, 574.3, 626.9, 696.8, 734.5, 773.7, 790.9, 819.5, 903.0, 916.0, 1021.9, 1113.9, 1195.2, 1221.3, 1255.4, 1277.5, 1291.7, 1331.6, 1365.3, 1412.4, 1463.3, 1821.3, 3205.2, 3329.8<br><br>B: 0.9288414, 0.6693781, 0.5261048	C 0.131847 0.072671 0.335612<br>C -0.053625 0.109809 1.808521<br>H -0.254719 -0.798441 2.353265<br>H -0.121043 1.069680 2.297611<br>C 1.349423 0.963626 -0.039755<br>C 0.330156 -1.373715 -0.173061<br>F 2.467679 0.408832 0.424978<br>F 1.209760 2.163372 0.524804<br>F 1.205041 -2.028809 0.586774<br>F 0.760960 -1.402607 -1.428782<br>F -0.832453 -2.026133 -0.123953<br>F 1.477871 1.138959 -1.345700<br>O -0.899724 0.675918 -0.474551<br>N -2.255359 0.521786 -0.088533<br>O -2.491558 -0.080234 0.912969<br>O -2.981624 1.049238 -0.858687
TS5<br><br>ν: -372.5, 38.5, 42.4, 56.2, 71.5, 93.4, 113.5, 151.3, 158.8, 212.4, 298.2, 313.3, 345.1, 372.4, 497.2, 503.1, 547.8, 554.1, 589.5, 647.8, 687.5, 694.7, 735.4, 797.2, 807.0, 868.6, 903.4, 948.1, 1061.7, 1121.5, 1202.9, 1220.8, 1237.4, 1251.6, 1290.3, 1388.5, 1414.4, 1451.5, 1621.4, 1728.1, 3194.1, 3299.0<br><br>B: 0.9515379, 0.5654986, 0.4371177	C -0.602714 -0.001638 0.564187<br>C 0.214151 -0.416641 1.561720<br>H 0.882007 0.286314 2.042295<br>H 0.078198 -1.394915 2.002886<br>C -0.506700 1.384214 -0.031073<br>C -1.601054 -0.938688 -0.074395<br>F 0.180945 1.363090 -1.174068<br>F 0.095515 2.225955 0.804027<br>F -1.544130 -0.863354 -1.402875<br>F -2.845954 -0.634059 0.302290<br>F -1.365856 -2.200566 0.284595<br>F -1.724899 1.861758 -0.298293<br>O 1.593878 -1.256514 0.223222<br>N 2.597946 -0.415058 -0.105794<br>O 3.429870 -0.945641 -0.783809<br>O 2.560188 0.711542 0.302044
P5<br><br>ν: 39.1, 46.8, 53.6, 70.5, 79.2, 145.0, 163.2, 211.3, 296.8, 310.2, 325.7, 343.1, 369.3, 483.0, 512.8, 548.8, 550.7, 614.5, 626.1, 660.1, 683.4, 751.0, 788.1, 807.0, 910.2, 933.3, 1031.9, 1090.8, 1170.0, 1184.1, 1204.8, 1226.3, 1272.6, 1297.6, 1373.9, 1387.2, 1403.0, 1446.3, 1479.8, 1807.6, 3102.8, 3174.5<br><br>B: 0.9254667, 0.6388049, 0.4846969	C -0.483053 -0.054122 0.532697<br>C 0.627661 0.289240 1.466188<br>H 0.630015 1.352130 1.700606<br>H 0.559787 -0.297230 2.385159<br>C -1.445247 0.965359 0.000186<br>C -0.497646 -1.423465 -0.075556<br>F -1.420531 1.008353 -1.332849<br>F -1.160087 2.183792 0.462911<br>F 0.387706 -1.509039 -1.080164<br>F -1.693981 -1.744200 -0.561504<br>F -0.155455 -2.339187 0.837460<br>F -2.700244 0.678714 0.370750<br>O 1.903130 -0.121824 0.949256

	N 2.291595 0.562043 -0.210088<br>O 3.337208 0.200143 -0.631362<br>O 1.539920 1.402035 -0.621103
Results from B3LYP/6-311++G(d,p) calculations	
$(CF_3)_2C=CH_2$<br><br>ν: 28.3, 60.2, 163.7, 196.0, 287.6, 312.0, 341.0, 360.7, 483.8, 489.6, 534.1, 536.2, 631.2, 671.3, 716.6, 759.0, 765.5, 865.5, 1019.5, 1084.6, 1115.6, 1127.8, 1158.6, 1192.6, 1231.7, 1357.7, 1449.9, 1720.6, 3170.5, 3263.1<br><br>B: 2.1266637, 1.0289765, 0.9192911	C -0.000000 0.000000 0.678300<br>C -0.000000 0.000000 2.005808<br>H -0.591561 -0.714695 2.562121<br>H 0.591561 0.714695 2.562121<br>C 0.799736 1.014086 -0.112079<br>C -0.799736 -1.014086 -0.112079<br>F 1.517843 0.433951 -1.093226<br>F 1.662722 1.682195 0.679654<br>F -0.000000 -1.933970 -0.691090<br>F -1.517843 -0.433951 -1.093226<br>F -1.662722 -1.682195 0.679654<br>F 0.000000 1.933970 -0.691090
NO_3 (D_{3h})<br><br>ν: 284.2, 284.4, 800.8, 1108.5, 1108.5, 1131.1<br>B: 13.8312308, 13.8312308, 6.9156154	O 0.000000 1.234075 0.000000<br>N 0.000000 0.000000 0.000000<br>O 1.068740 -0.617038 0.000000<br>O -1.068740 -0.617038 0.000000
TS4<br><br>ν: -296.4, 20.7, 36.0, 48.3, 65.6, 113.5, 148.8, 163.0, 201.2, 264.8, 285.8, 305.3, 330.4, 348.5, 447.4, 487.0, 525.0, 540.2, 573.8, 583.9, 623.4, 657.8, 677.5, 696.4, 767.7, 773.3, 865.9, 900.7, 951.7, 1079.0, 1107.5, 1150.2, 1181.1, 1186.1, 1221.5, 1290.6, 1331.4, 1421.5, 1530.2, 1611.4, 3176.8, 3282.8<br><br>B: 0.8640336, 0.6091140, 0.4779168	C -0.401277 0.000008 0.480733<br>C 0.029422 -0.000002 1.789669<br>H 0.231609 0.928931 2.303951<br>H 0.231505 -0.928948 2.303969<br>C -0.956782 -1.311235 -0.084289<br>C -0.956589 1.311315 -0.084326<br>F -2.225416 -1.480581 0.352044<br>F -0.234593 -2.353171 0.363878<br>F -2.225179 1.480891 0.352046<br>F -0.977611 1.353600 -1.414629<br>F -0.234212 2.353151 0.363770<br>F -0.977763 -1.353574 -1.414591<br>O 1.245946 -0.000172 -0.661083<br>N 2.487697 -0.000101 -0.099760<br>O 2.608811 0.000023 1.107714<br>O 3.358658 -0.000179 -0.939503
P4<br><br>ν: 33.6, 45.8, 72.0, 81.1, 147.4, 169.0, 210.5, 226.3, 240.5, 278.1, 296.1, 316.0, 336.0, 346.6, 369.1, 487.2, 528.8, 534.4, 555.2, 579.4, 604.0, 662.0, 687.8, 737.3, 745.2, 786.3, 811.5, 878.3, 933.7, 1077.3, 1134.0, 1148.5, 1170.4, 1193.4, 1207.4, 1253.1, 1296.8, 1339.8, 1454.8, 1756.5, 3178.6, 3299.7<br><br>B: 0.9000331, 0.6546159, 0.5094836	C 0.139465 0.056292 0.330822<br>C -0.082012 0.090556 1.798290<br>H -0.231755 -0.821307 2.354166<br>H -0.163530 1.047249 2.292650<br>C 1.329010 1.016872 -0.032378<br>C 0.393412 -1.396230 -0.173995<br>F 2.471800 0.563714 0.506988<br>F 1.084995 2.237334 0.475644<br>F 1.366972 -1.990847 0.534890<br>F 0.734321 -1.433076 -1.466131<br>F -0.727015 -2.127013 -0.025897<br>F 1.511371 1.148571 -1.347295<br>O -0.916482 0.622730 -0.504337<br>N -2.318084 0.481870 -0.090951<br>O -2.552997 -0.124348 0.915406<br>O -3.035444 1.027604 -0.867367

TS5<br><br>ν: -364.6, 30.1, 38.9, 44.7, 48.7, 62.6, 118.7, 150.4, 161.5, 213.1, 291.5, 306.3, 335.0, 363.0, 479.0, 492.3, 534.0, 540.9, 570.9, 629.9, 664.1, 672.9, 710.6, 764.9, 775.8, 843.7, 862.4, 906.4, 1062.5, 1094.6, 1110.0, 1134.5, 1156.3, 1194.6, 1202.5, 1290.2, 1352.4, 1422.2, 1543.5, 1608.5, 3180.2, 3281.3<br><br>B: 0.9369232, 0.5042713, 0.3926242	C -0.628710 -0.027030 0.490897<br>C 0.340087 -0.411489 1.381428<br>H 0.962151 0.337633 1.849980<br>H 0.290710 -1.389821 1.837147<br>C -0.690955 1.390104 -0.043211<br>C -1.634354 -1.023480 -0.048372<br>F -0.080308 1.484191 -1.239605<br>F -0.085089 2.247917 0.794743<br>F -1.755814 -0.932822 -1.382568<br>F -2.852709 -0.813718 0.491235<br>F -1.274177 -2.283665 0.251372<br>F -1.962937 1.797020 -0.200544<br>O 1.778262 -1.126751 0.130383<br>N 2.842168 -0.326243 -0.094330<br>O 3.708836 -0.894435 -0.727056<br>O 2.842261 0.805804 0.328801
P5<br><br>ν: 314.2, 333.1, 345.6, 459.1, 499.0, 532.9, 537.9, 558.5, 601.8, 631.6, 642.9, 717.1, 757.4, 761.8, 840.6, 896.0, 969.4, 1045.7, 1081.4, 1111.6, 1125.4, 1143.4, 1194.4, 1269.4, 1317.9, 1330.0, 1335.1, 1410.2, 1456.8, 1740.7, 3049.4, 3140.1<br><br>B: 0.9083522, 0.5844595, 0.4458841	C -0.468618 -0.008436 0.419903<br>C 0.649246 0.316006 1.358540<br>H 0.714321 1.387931 1.533987<br>H 0.512356 -0.199008 2.314955<br>C -1.468772 1.027495 -0.012668<br>C -0.612874 -1.418354 -0.086497<br>F -1.631964 1.039218 -1.347886<br>F -1.090298 2.261950 0.367501<br>F 0.231312 -1.655723 -1.116113<br>F -1.855447 -1.688812 -0.513899<br>F -0.311777 -2.301749 0.889568<br>F -2.681977 0.797217 0.541458<br>O 1.922652 -0.210328 0.929654<br>N 2.500219 0.487771 -0.198514<br>O 3.550413 0.027241 -0.523353<br>O 1.869342 1.411525 -0.646384
